# Impaired Mobilization of Vascular Reparative Bone Marrow Cells in Streptozotocin-Induced Diabetes but not in Leptin Receptor-Deficient db/db Mice

**DOI:** 10.1038/srep26131

**Published:** 2016-05-18

**Authors:** Goutham Vasam, Shrinidh Joshi, Yagna P. R. Jarajapu

**Affiliations:** 1 Department of Pharmaceutical Sciences, North Dakota State University, Fargo, North Dakota, USA

## Abstract

Diabetes is associated with impaired mobilization of bone marrow stem/progenitor cells that accelerate vascularization of ischemic areas. This study characterized mobilization of vascular reparative bone marrow progenitor cells in mouse models of diabetes. Age-matched control or streptozotocin (STZ)-induced diabetic, and db/db mice with lean-controls were studied. Mobilization induced by G-CSF, AMD3100 or ischemia was evaluated by flow cytometric enumeration of circulating Lin^−^Sca-1^+^cKit^+^ (LSK) cells, and by colony forming unit (CFU) assay. The circulating WBCs and LSKs, and CFUs were reduced in both models with a shorter duration (10–12 weeks) of diabetes compared to their respective controls. Longer duration of STZ-diabetes (≥20 weeks) induced impairment of G-CSF- or AMD3100-mobilization (P < 0.01, n = 8). In db/db mice, mobilization by G-CSF or AMD3100 was either increased or unaffected (P < 0.05, n = 6 to 8). Proliferation, migration, and ischemia-induced mobilization, of LSK cells were impaired in both models. Leptin receptor antagonist, PESLAN-1, increased G-CSF- or AMD3100-mobilization of WBCs and LSKs, compared to the untreated. Leptin increased basal WBCs, decreased basal and AMD3100-mobilized LSK cells, and had no effect on G-CSF. These results suggest that mobilopathy is apparent in STZ-diabetes but not in db/db mice. Leptin receptor antagonism would be a promising approach for reversing diabetic bone marrow mobilopathy.

Evidence has been accumulating for the therapeutic efficacy of different populations of bone marrow progenitor cells for the treatment of ischemic cardiovascular diseases. Compelling evidence has been provided for the angiogenic propensity of bone marrow-derived cells, often termed as endothelial progenitor cells, which stimulate re-endothelialization and vascular regeneration in experimental and preclinical studies^1^,^2^,^3^,^4^,^5^. It is now known that vasoreparative cells are mobilized from bone marrow niches into the blood circulation in response to vascular injury, migrate to the areas needing repair and accelerate vascular repair^3^. Therefore stem/progenitor cells offer a breakthrough approach for the treatment of cardiovascular disease.

Mobilization and harvesting of cells is a key determinant of the success of autologous cell therapies. Agents such as granulocyte-colony stimulating factor (G-CSF) and AMD3100 that are well known for their efficient mobilization of stem/progenitor cells, have been shown to accelerate ischemic vascular repair in experimental models of ischemic injury^2^,^6^,^7^,^8^,^9^. Therefore these mobilizers are potentially useful for mobilization and collection of EPCs in adequate numbers required for autologous cell therapies for cardiovascular disease.

Diabetes, either type 1 or type 2, increases risk for cardiovascular disease. Patients with diabetes are most suitable for autologous cell therapies however several challenges have yet to overcome. Clinical studies have shown that diabetes is associated with a reduced number of circulating stem/progenitor cells, which consequently results in tremendous decrease in the innate vasoreparative propensity following ischemic injury^10^,^11^,^12^. Therefore autologous cell-based therapies are currently not feasible for the treatment of diabetic vascular disease. These findings have been recapitulated in a few experimental studies as follows. Streptozotocin (STZ)-induced diabetes is an extensively used mouse model for type 1 diabetes, whereas leptin or leptin receptor (Lepr)-deficient mice, ob/ob and db/db respectively, are frequently used as models of type 2 diabetes. Diabetic stem cell mobilopathy has been demonstrated in STZ-diabetic mice with duration of diabetes as short as 8-12 weeks^13^,^14^ however studies in type 2 diabetic models are limited.

This study characterized mobilization of vasoreparative bone marrow stem/progenitor cells in STZ- and db/db-mouse models of diabetes in response to G-CSF or AMD3100, and in response to ischemic injury. In mice, Lineage-negative, Sca-1^+^ and cKit^+^ (LSK) cells are vasculogenic and accelerate vascular repair^16^,^17^,^18^. LSK cell population is mostly consist of hematopoietic progenitor cells with ~10% stem cells^15^. This cell population has the highest potential of endothelial cell lineage development with vasoreparative functions, while LS or LK cells have comparable vasculogenic functions, and are mobilized in response to ischemic injury^16^,^17^,^18^. Our findings showed impairment of LSK mobilization in mice with STZ-induced diabetes in response to all stimuli, but not in db/db mice. G-CSF- or AMD3100-induced mobilization was either enhanced or unchanged in type 2 diabetic db/db mice compared to their lean nondiabetic control mice. This was further confirmed by using a pharmacological antagonist of Lepr in wild type mice. Collectively, our findings propose a role of Lepr in mobilization of stem/progenitor cells, and lead to the hypothesis that antagonism of Lepr is a promising target for reversing diabetic impairment of mobilization.

## Results

### Circulating LSK cells are reduced in STZ- and db/db mice

**Figure 1:**
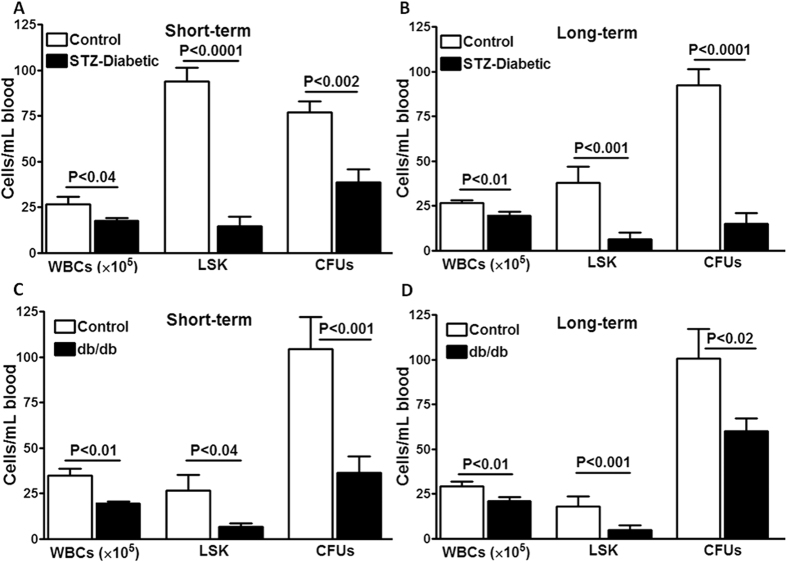
Experimental diabetes is associated with decreased number of circulating WBCs and LSK cells. In STZ-mice with short-term diabetes (10–12 weeks) (**A**) or long-term diabetes (≥20 weeks) (**B**), number of WBCs or LSK cells (n = 8), and CFUs derived from circulating blood (n = 6 to 10), were lower compared to age-matched non-diabetic controls. Similar changes were observed in db/db mice with short-term diabetes (10–12 weeks) (**C**) or long-term diabetes (≥20 weeks) (**D**).

In STZ-mice, circulating WBCs and LSK cells were decreased by as early as 10–12 weeks of diabetes compared to the age-matched controls (WBCs P < 0.04, LSK cells P < 0.0001, n = 8) (Fig. 1A). This decrease was further exacerbated with longer duration of diabetes (≥20 weeks) (WBCs P < 0.01, LSK cells P < 0.001, n = 8 to 12) (Fig. 1B). Consistent with the flow cytometric enumeration, CFUs derived from peripheral blood were decreased in STZ-diabetes compared to control (n = 6 to 10) (Fig. 1A,B). Similar changes were observed in db/db mice with short-term or long-term diabetes compared to lean-control mice. In db/db mice with 10–12 weeks of diabetes, circulating WBCs (P < 0.01) and LSK cells (P < 0.04, n = 6) were reduced (Fig. 1C), which was further exaggerated with longer duration of diabetes (WBCs P < 0.01, LSK cells P < 001, n = 10) (Fig. 1D). In agreement with the flow cytometric enumeration, the number of CFUs was decreased in the peripheral blood of db/db mice compared to lean mice (n = 6 to 10) (Fig. 1C,D).

### Drug-induced mobilization of bone marrow cells is differentially affected in STZ- and db/db mice

**Figure 2:**
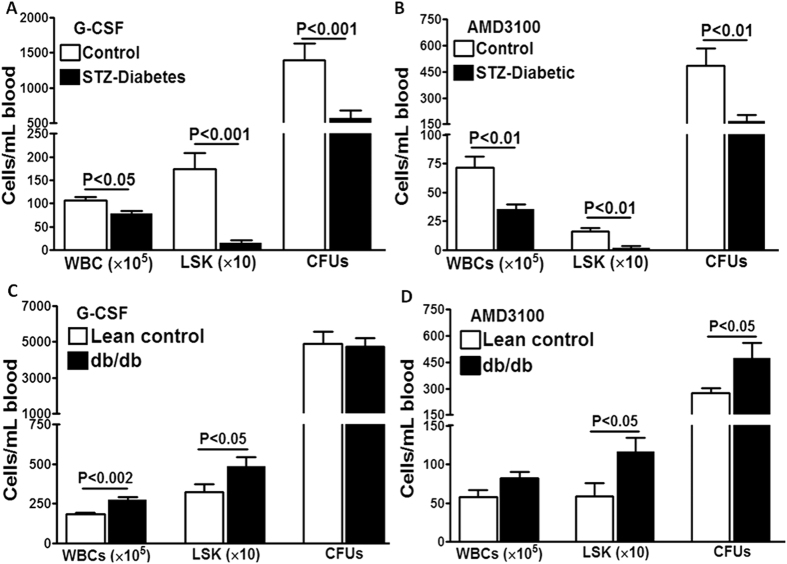
Mobilization by G-CSF or AMD3100 is impaired in STZ-diabetes but not in db/db mice. G-CSF- (**A**) or AMD3100-induced (**B**) mobilization of WBCs or LSK cells (n = 8) and CFUs derived from mobilized blood cells (n = 6) were decreased in STZ-mice with long-term diabetes (≥20 weeks) compared to controls. G-CSF- (**C**) or AMD3100-induced (**D**) mobilization of WBCs or LSK cells (n = 8) and CFUs were increased in db/db mice compared to lean-controls (n = 6).

Then, we checked mobilization of bone marrow cells induced by G-CSF or AMD3100 in STZ- and db/db mice and compared with their respective controls. In STZ-mice with short-term diabetes, mobilization of LSK cells to either AMD3100 or G-CSF was not affected (data not shown). Mobilization of WBCs or LSK cells was impaired in STZ mice with long-term diabetes in response to G-CSF (WBCs P < 0.05; LSK cells P < 0.001, n = 8) (Fig. 2A) or AMD3100 (WBCs P < 0.01, LSK cells P < 0.01, n = 8) compared to the age-matched controls (Fig. 2B). Accordingly, the CFUs derived from G-CSF or AMD3100-mobilized cells were lower in STZ-diabetes compared to the controls (P < 0.001 and P < 0.01, respectively, n = 6) (Fig. 2A,B). Thus, STZ-diabetes recapitulated bone marrow mobilopathy that is observed in individuals with diabetes.

In contrast, and to our surprise, mobilization of LSK cells to either G-CSF or AMD3100 was either enhanced or unchanged in db/db mice compared with the age-matched lean-control mice, regardless of the age or duration of diabetes. G-CSF-mobilized WBCs or LSK cells were higher compared to lean-control mice (P < 0.002 and P < 0.05, respectively, n = 8) with similar number of CFUs in both groups (Fig. 2C). In response to AMD3100, WBCs, LSKs and CFUs were higher in db/db mice (P < 0.05 for all parameters, n = 6 to 8) compared to the age-matched lean-controls (Fig. 2D).

It is important to note that compared to the control mice (control for STZ-diabetes), mobilization of LSK cells was 2- and 3-fold higher in lean-control and db/db mice, respectively, in response to G-CSF. Along the similar lines, LSK mobilization was 2- and 5-fold higher in response to AMD3100 in the lean control and db/db mice, respectively. These results suggested that in the absence of functional Leprs, as in db/db mice (Lepr^−/−^), mobilization to AMD3100 or G-CSF is either increased or rescued from diabetic mobilopathy. Together with results in lean mice that are Lepr^+/−^, these studies suggest that complete loss of functional Leprs is needed for causing type 2 diabetes, whereas, partial loss of the functional receptors would be sufficient to rescue mobilization of bone marrow cells to AMD3100 or G-CSF from diabetic dysfunction.

**Figure 3:**
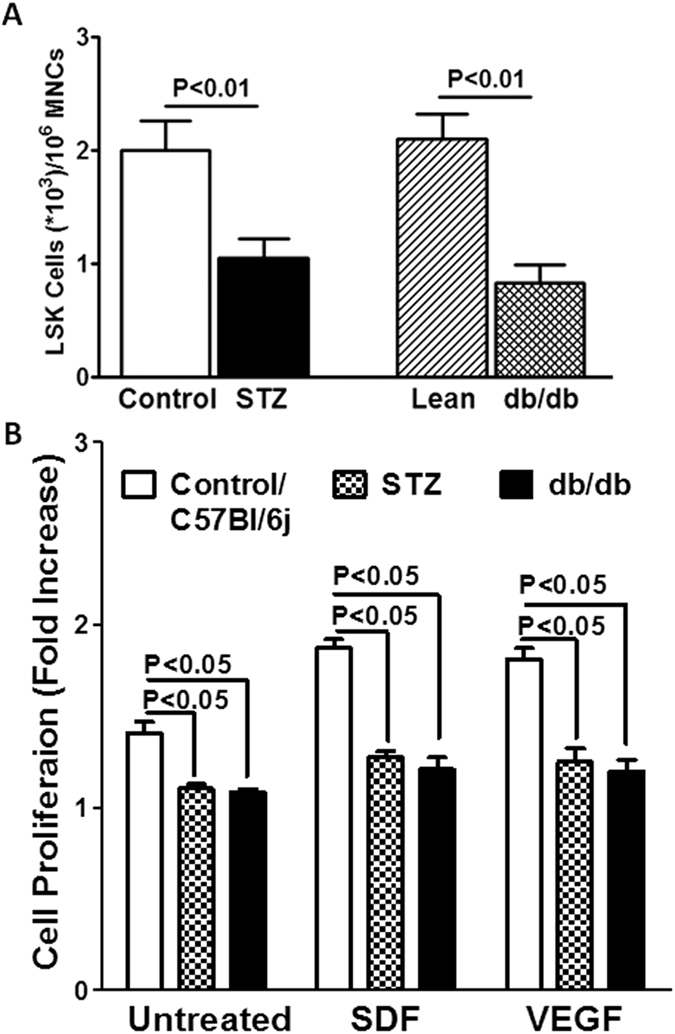
Experimental diabetes decreases the number of bone marrow LSK cells. (**A**) Number of LSK cells in the bone marrow are decreased in STZ and db/db mice compared to the respective control mice (n = 6). (**B**) Proliferation of bone marrow-derived LK cells was attenuated in STZ-diabetic or db/db mice compared to controls, in the basal conditions or in response to treatment with either stromal-derived factor-1α (SDF) or vascular endothelial growth factor (VEGF) (n = 5).

Then, we asked if this exacerbated responses to G-CSF or AMD3100 in Lepr-deficient mice could be due to increased number of bone marrow-resident progenitor cells. To answer this, LSK cells were enumerated in the whole bone marrow of STZ and db/db mice, and their age-matched lean-controls. Number of LSK cells (P < 0.002, n = 6) in the bone marrow was decreased in both models compared to their respective age-matched controls (P < 0.01 Fig. 3A) suggesting that diabetes is associated with depletion of vascular reparative progenitor cells. This was further confirmed by determining proliferative propensity of bone marrow LK cells. *Ex vivo* proliferation of LK cells from control mice for STZ-diabetes was similar to that of lean-control mice (data not shown). Proliferative propensity of LK cells was attenuated in STZ-diabetes (P < 0.05, n = 6) or in db/db mice (P < 0.05, n = 5) compared to control (Fig. 3B). This dysfunction was also observed in response to hypoxia-regulated factors, stromal-derived factor-1α (SDF) (P < 0.05, n = 5) or vascular endothelial growth factor (VEGF) (Fig. 3B) (P < 0.05, n = 5).

**Figure 4:**
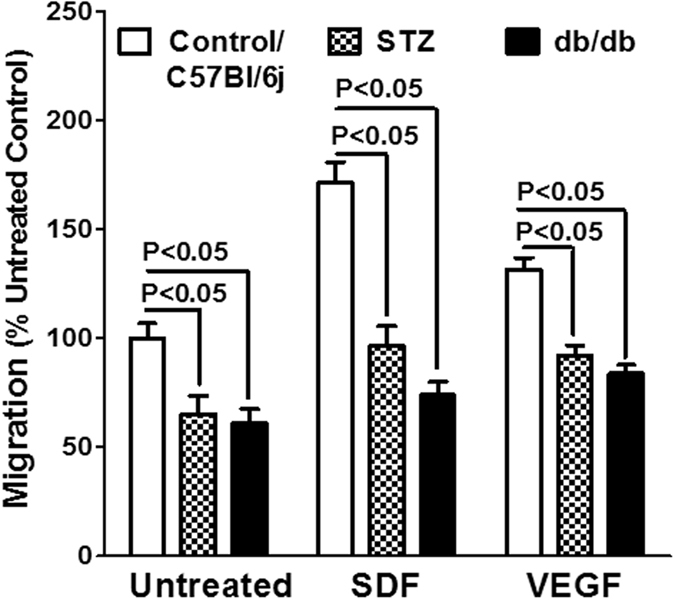
Experimental diabetes decreases the migratory function of bone marrow cells. Migration of bone marrow LK cells was decreased in the basal or in response to SDF or VEGF in both STZ-diabetic and db/db mice (n = 5).

SDF and VEGF are potent chemoattractants of bone marrow stem/progenitor cells, and elevated levels of these factors in the systemic circulation would stimulate mobilization of these cells^19^,^20^. SDF has been implicated directly or indirectly in the mobilization induced by either G-CSF or AMD3100^21^. Therefore we asked if the migratory propensity of stem/progenitor cells to SDF or VEGF, is higher in Lepr-deficient mice, which may account for the differences observed in G-CSF or AMD3100. *In vitro* migration assays showed that migration to either SDF- or VEGF-gradient were similar in control mice for STZ-diabetes and Lepr^+/−^ lean mice (data not shown), and furthermore this response was impaired in LK cells derived from STZ-diabetic mice (P < 0.05, n = 6) or db/db mice compared to non-diabetic controls. (P < 0.05, n = 6) (Fig. 4). Furthermore, circulating levels of SDF and VEGF were not different in Lepr-deficient mice compared to control or STZ-diabetic mice (Table 1). In addition, we evaluated the plasma levels of IGF-1 and IGFBP3 that were shown to induce stem/progenitor cell mobilization in different experimental models^22^,^23^. IGFBP3 levels were similar among different experimental groups however IGF-1 levels were higher in db/db mice compared to wild type or lean mice (P < 0.03, n = 6) (one-way ANOVA, Bonferroni’s post-test) (Table 1).

### Ischemia-induced mobilization is impaired in both STZ- and db/db mice

**Figure 5:**
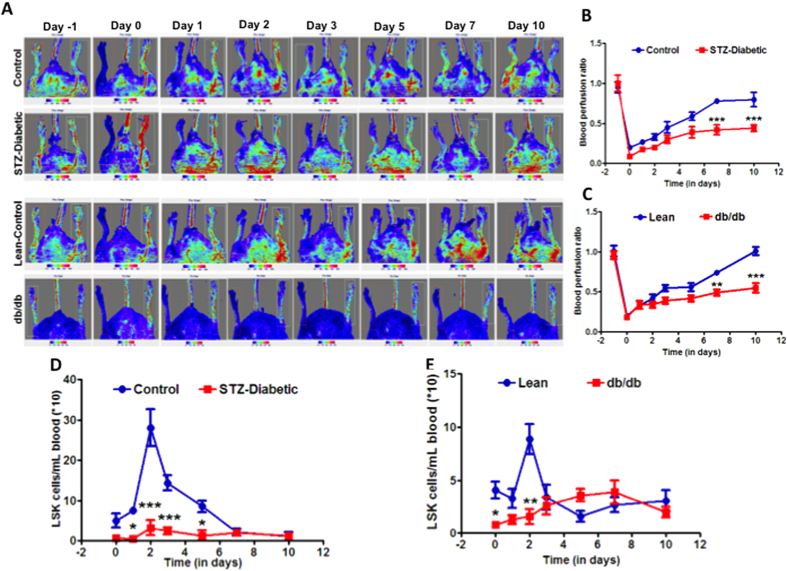
Experimental diabetes decreases mobilization of LSK cells and blood flow recovery following hind-limb ischemia. **(A)** Pseudo-color images of flux of blood in mice before and after ischemia. Blood perfusion to the ischemic limb was expressed as ratio of the blood flow to the contralateral limb. Recovery of blood flow was significantly decreased in STZ-diabetic mice (n = 6) (**B**), and in db/db mice (n = 6) (**C**) compared to their respective controls mice (n = 6). (**D)** LSK mobilization following ischemia was decreased in STZ-diabetic mice on days, 1, 2, 3, and 5 compared to controls (n = 6). (**E**) In db/db mice, this decrease was observed on day 2 following ischemia (n = 6). Data sets were analyzed by two-way ANOVA with Bonferroni’s post-test.

Then, we checked if ischemia-induced mobilization is different in these two models of diabetes. In control mice, HLI by femoral ligation reduced blood flow to almost 10% of contralateral limb, which was gradually restored starting from the day 2, and 70% recovery was observed by day-10 following HLI (Fig. 5A,B). Ischemic injury induced mobilization of LSK cells resulting in 10–fold increase in the number of circulating cells compared to pre-ischemic levels by day-2, which remained significantly higher until day-7, and returned to pre-ischemic levels by day 10 (Fig. 5D). In STZ-mice with 10–12 weeks of diabetes, maximum blood flow recovery at day-10 post-HLI was 47%, which is lower than the recovery observed in the control group (P < 0.0001, n = 6) (Fig. 5A,B). Mobilization of LSK cells was not observed in STZ mice on days-1, 2, 3 and 5 that was apparent in the control mice (n = 6) (Fig. 5D).

Similar dysfunction was observed in db/db mice with 10–12 weeks of diabetes compared to lean-control mice. Blood flow restoration was almost 100% following HLI in lean-control while the recovery was only 53% in db/db mice (P < 0.001, n = 6) (Fig. 5A,C). In lean-controls, HLI-induced LSK mobilization was 3-fold higher compared to pre-ischemic levels by day 2 (n = 6), which returned to pre-ischemic levels by day-3 in lean mice. (Fig. 5E). In db/db mice, LSK mobilization was not observed until day-3 and remained higher until day-7 and then returned to pre-ischemic levels by day-10. However this increase in LSK cells was significantly lower than that observed in lean control mice (P < 0.01, n = 6) (Fig. 5E). Therefore in both models of diabetes, regardless of Lepr-deficiency, ischemia-induced mobilization of LSK cells and blood flow recovery to ischemic area were impaired with a shorter duration of diabetes.

### Lepr antagonist robustly increased mobilization of bone marrow cells to G-CSF or AMD3100

**Figure 6:**
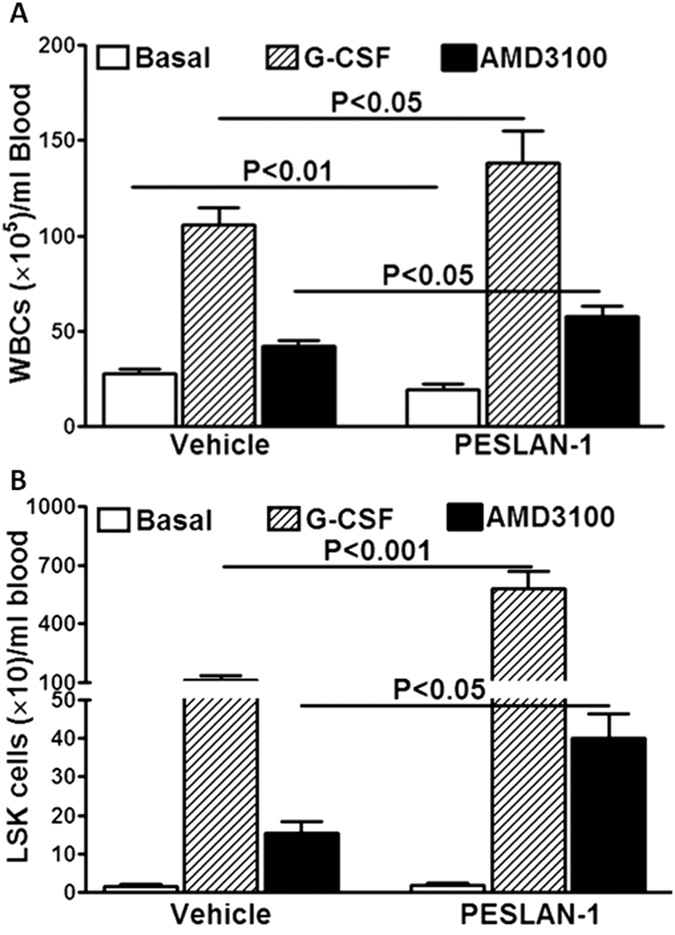
Leptin receptor antagonist PESLAN-1 potentiates G-CSF or AMD3100 mobilization. **(A)** PESLAN-1 decreased basal (n = 7), and strongly potentiated G-CSF (n = 7) or AMD3100-induced mobilization of WBCs (n = 6). **(B)** Pretreatment with PESLAN-1 potentiated LSK-mobilization by G-CSF (n = 7) or AMD3100 (n = 6) with no change in basal levels.

Then, we tested if pharmacological antagonism of LepR would recapitulate the findings in Lepr-deficient mice on AMD3100 or G-CSF-induced mobilization. This was accomplished by using a peptide antagonist of Lepr, PESLAN-1^24^ in wild type mice. PESLAN-1 caused gradual increase in body weight, and the weight gain was plateaued by day-14 with a maximum weight gain of 14% (3.5 grams) relative to the untreated control mice on similar diet with no significant change in the blood glucose levels. The weight gain was readily reversible following cessation of the treatment. PESLAN-1 treatment decreased circulating WBCs (P < 0.05, n = 8, Fig. 6A) but has no effect on LSK cells (Fig. 6B). PESLAN-1 pretreatment resulted in robust potentiation of G-CSF-mobilization of WBCs (P < 0.05) or LSK cells (P < 0.001, n = 7) compared to the vehicle treatment (Fig. 6B,C). Similar findings were observed on WBCs (P < 0.05, n = 6) or LSK cells (P < 0.05, n = 6) mobilized by AMD3100 (Fig. 6A,B).

### Leptin differentially affects physiological, and induced mobilization of bone marrow cells

**Figure 7:**
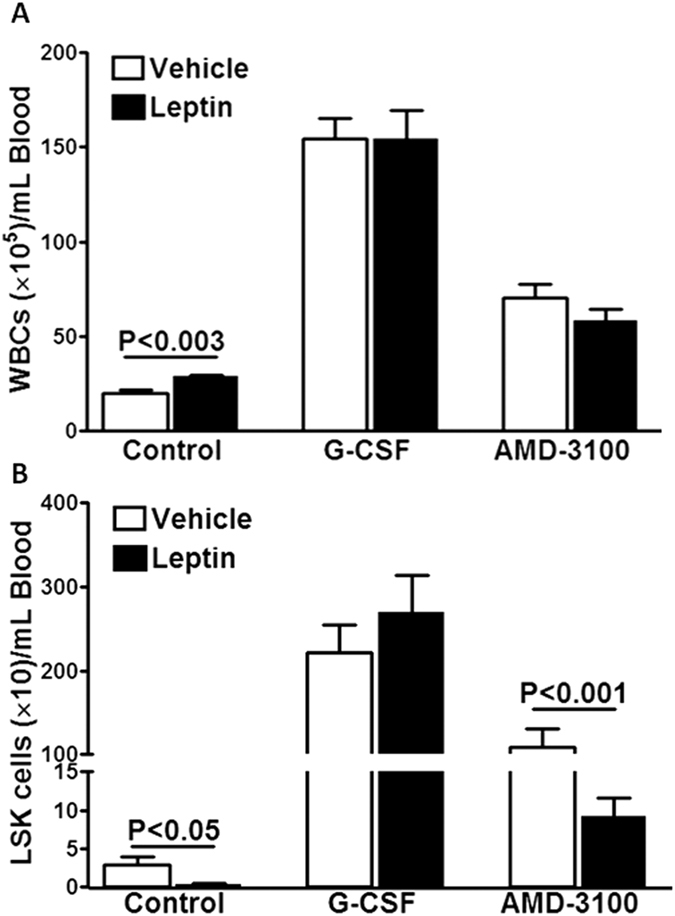
Leptin differentially affects mobilization of WBCs and LSK cells. (**A)** Leptin increased basal WBC levels (n = 8) and has no effect on G-CSF- (n = 8) or AMD3100- (n = 6) induced WBC mobilization. (**B)** Leptin reduced basal (n = 8) and AMD3100-induced (n = 6) LSK mobilization while G-CSF mobilization was unaffected (n = 6).

Lastly, we have evaluated the effects of leptin treatment on stem/progenitor cell mobilization. Treatment with leptin in mice resulted in gradual decrease in body weight and 11% (compared to the weight on day-0) decrease was observed by day-6. Consistent with several previous reports, leptin treatment increased the circulating WBCs (P < 0.003, n = 8, Fig. 7A), however, interestingly decreased LSK cells (P < 0.05, n = 8, Fig. 7B). Then, we tested if leptin treatment alters the mobilization induced by G-CSF or AMD3100. G-CSF caused robust mobilization of WBCs and LSK cells. Leptin did not affect G-CSF mobilization of WBCs or LSK cells (n = 8, Fig. 7A,B). AMD3100-induced mobilization of LSK cells (P < 0.001, n = 6) but not WBCs, was reduced by leptin (Fig. 7A,B).

## Discussion

This study reports several novel findings. 1. In both STZ and db/db models of diabetes, mobilization of bone marrow cells is impaired at as early as 10–12 weeks of diabetes, 2. Drug-induced mobilization, either by AMD3100 or G-CSF was not affected in the early stages of diabetes and required longer duration of diabetes in the STZ-model, 3. Despite severe diabetes, mobilization to G-CSF or AMD3100 was robustly increased in mice with genetic ablation of lepr, db/db mice or their lean controls, 4. Pharmacological antagonism of leptin receptor by PESLAN-1 exacerbates mobilization to G-CSF or AMD3100, 5. Treatment with leptin increased circulating WBCs but reduced the number of LSK cells. Leptin did not affect G-CSF mobilization however reduced LSK mobilization by AMD3100, and 6. Both STZ- and db/db mouse models of diabetes are suitable for studying diabetic dysfunction in vascular repair and ischemia-induced mobilization of bone marrow progenitor cells. However STZ-, not Lepr-deficient, model of diabetes is useful for studying dysfunctional mobilization to G-CSF or AMD3100. Importantly, leptin receptor antagonism is a promising approach for reversing diabetic bone marrow mobilopathy.

It is important to note that pharmacological concentrations of exogenous leptin increased basal levels of WBCs while decreasing LSK cells, however pharmacological antagonism of physiological leptin has no effect on basal levels of LSK cells though reduced WBCs. This is in agreement with the lymphopoietic/myelopoietic effects of leptin that could be antagonized by PESLAN-1 and that leptin does not induce mobilization of LSK cells. *In vitro* studies have shown that leptin promotes hematopoiesis via its direct actions on hematopoietic stem cells^25^,^26^,^27^ at high concentrations of 100 ng/ml, and at physiological concentrations this effect was not observed^27^,^28^. In obese subjects circulating leptin levels could reach higher than 100ng/ml, and the leptin levels positively correlate with the number of circulating leukocytes^27^. In leptin-deficient ob/ob mice, lymphopoiesis is reduced that could be reversed by exogenous leptin supplementation^25^,^29^. On the other hand, in Lepr-deficient mice both lymphopoiesis and myelopoiesis were impaired^25^. These studies indicate that Lepr is involved in hematopoiesis induced by other factors while leptin itself has limited pro-hematopoietic functions. In addition, concentrations of leptin that induced proliferation *in vitro* are pathological as shown by Chung *et al.*^28^. In T2D patients with nephropathy, leptin levels positively correlated with total leukocyte counts^28^. It is important to note that proliferation of progenitor cells alone is not sufficient to induce mobilization as shown previously by using cyclophosphamide or IL-8 in G-CSFR KO mice^30^. Therefore the observations in the current study are independent of hematopoietic effects of leptin.

Ob-Rb isoform that encodes full-length LepR, including the intracellular signaling domain mediates physiological effects of leptin. The loss of the full-length LepR confers defective leptin signaling in db/db mice, which express the truncated splice variants of the receptor^31^. Functional Leptin-Lepr system is expressed in several tissues including bone marrow. Very recent studies provided compelling evidence for the Lepr as an excellent marker for the prospective identification of murine mesenchymal stromal cells (MSCs)^32^,^33^. Lepr^+^ MSCs regulate quiescence and retention of stem cells in bone marrow via paracrine signaling. Findings from the current study suggest that pharmacological or genetic disruption of leptin-lepr interaction would be sufficient to sensitize the stem/progenitor cells for mobilization by agents such as G-CSF or AMD3100.

While there is no consensus in regards to the mechanism of G-CSF mobilization, G-CSF and AMD3100 act via distinct mechanisms. AMD3100 acts by blocking CXCR4 on stem/progenitor cells, which makes them nonresponsive to SDF resulting in disruption of the retention^34^. An alternative mechanism of AMD3100 mobilization is based on the assumption that it acts on CXCR4-expressing MSCs. Dar *et al.*^35^ have shown that AMD3100 stimulate MSCs to secrete SDF into peripheral blood thus reversing the retention gradient in favor of mobilization. However no explanation has been provided for its preferential binding to CXCR4 receptor on MSCs over HSCs. On the other hand, several mechanisms have been proposed to explain G-CSF mobilization. G-CSF increases proliferation and differentiation, and causes egress by altering bone marrow architecture. Mechanisms include and not limited to increased protease levels, leakage of blood vessels, osteoblast suppression, and partial disruption of VCAM/VLA4 as well as CXCR4/SDF interactions^21^. Despite the diverse mechanisms involved, mobilization by G-CSF or AMD3100 could be augmented by pharmacological blockade of Lepr, which suggests that an important retention signaling is provided by leptin-Lepr interaction in the bone marrow.

The study by Ferraro *et al.*^13^ reported decreased basal levels of LSK cells in both STZ and db/db models of diabetes, and impaired G-CSF mobilization in STZ diabetes. Previous studies have demonstrated increased mobilization of LSK cells to AMD3100 in db/db and vascular regeneration in ischemic areas^17^,^36^. However these studies concluded that AMD3100 is effective mobilizer in diabetes, and did not investigate the influence of deficient Lepr-signaling. G-CSF mobilization has been shown to be strain-dependent, and C57Bl/6j was shown to be a poor mobilizer to G-CSF or AMD3100 34,37 therefore genetic differences in the background strain could be one reason for the exacerbated mobilization response to G-CSF in lean or db/db mice. This possibility was however ruled out in the current study by using a pharmacological antagonist of leptin receptor, which recapitulated the observations in the genetic model of LepR dysfunction in C57Bl/6j. Therefore collectively these findings lead to the conclusion that the physiological leptin-Lepr signaling antagonizes G-CSF or AMD3100 mobilization of bone marrow cells. Furthermore, the present study shows that LepR antagonist would enhance mobilization to G-CSF or AMD3100 in healthy conditions, and would reverse diabetic dysfunction of mobilization independent of diabetic profile as long as functional LepRs are present.

In the current study no changes in the circulating cytokines or growth factors such as SDF, VEGF, or IGFBP3 was observed, the gradient of which were shown to induce mobilization^17^,^19^,^22^,^23^. In particular SDF has been well studied for its role in mobilization and mostly involves reversal of concentration gradient i.e. decreased levels in bone marrow relative to the circulating SDF levels^21^. Ferraro *et al.*^13^ found no change in bone marrow SDF levels despite decreased circulating LSK cells in STZ or db/db mice, which is in agreement with the current study. Decreased number of circulating LSK cells despite physiological levels of circulating and bone marrow SDF levels suggested impaired mobilization signaling of SDF. As shown in the current study migration of progenitor cells to SDF or VEGF is impaired in diabetes, which is in consistent with previous studies in human stem cells derived from diabetic human individuals^17^,^38^,^39^. Thus, despite diabetes-induced migratory defect, db/db mice could mobilize cells in response to G-CSF or AMD3100 but not in STZ-diabetic mice, which further supports the notion that lack of inhibitory signal from leptin-Lepr signaling preserves or even augments mobilization. Interestingly, IGF-1 levels were increased in Lepr^−/−^ db/db mice compared to Lepr^+/−^ as well as Lepr^+/+^ mice. It is important to note that insulin levels are also higher in db/db mice^40^ however neither of these explain the differences in the augmented mobilization, which is also observed in Lepr^+/−^ mice with no changes in the circulating levels of either of these factors.

Diabetic dysfunction of ischemia-induced mobilization is observed in both models of diabetes with relatively shorter duration, 10–12 weeks, of diabetes. This is consistent with the *ex vivo* studies showing evidence for impaired migration to SDF and VEGF, which are hypoxia-regulated factor with potent chemoattractant properties for stem cell mobilization and recruitment to areas of ischemia, and *in vivo* studies in STZ-diabetic and db/db mouse models^17^,^41^,^42^,^43^. Tepper *et al.*^17^ showed impaired LSK cell mobilization in response to cutaneous wound and another study by Yan *et al.*^43^ showed impaired mobilization of CD34^+^, flk1^+^ or CD133^+^ cells in db/db mice on day-7 following HLI. De Falco *et al.*^42^ evaluated mobilization of LK cells longitudinally with blood flow recovery in small group of STZ-diabetic mice and reported impaired mobilization of LK cells only at day-7 following HLI. Similar dysfunction has been observed in a mouse model of high-fat diet-induced diabetes^44^,^45^ Current study showed that mobilization of LSK cells is impaired up to day-5 in STZ-diabetes. Interestingly, the mobilization was observed only at day-2 post-HLI in lean control mice, which was impaired in db/db mice. It is important to note that the extent of mobilization is only 2 to 3- fold in lean control mice compared to 5 to 6-fold in C57Bl/6 mice, suggesting that Lepr-deficiency, at least in part, modulates stem/progenitor cell mobilization in response to vascular injury, which needs further investigation.

This study does not address cellular and molecular signaling mechanisms that mediate the interaction of leptin or Lepr antagonist on G-CSF or AMD3100 mobilization. Speculatively, interaction would be most likely at the level of Lepr^+^ bone marrow mesenchymal stromal cells, which are now known to maintain retention, quiescence and maintenance of stem cells by secreting SDF and SCF^32^,^46^. However possible interactions cannot be ruled out at bone marrow endothelial cells, osteoblasts, neutrophils, or macrophages^47^,^48^,^49^. A systematic mechanistic study is needed for understanding these cellular and molecular interactions.

In conclusion, our study provides compelling evidence for a novel role of lepr signaling in the regulation of stem/progenitor cell mobilization by G-CSF or AMD3100, and proposes that Lepr blockade as a novel approach for reversing stem cell mobilopathy in poor mobilizers^50^,^51^ or in diabetic individuals, which increases the success rate of cell-based therapies for diabetic cardiovascular disease or in patients undergoing chemo/radiation therapy. Exploring the therapeutic utility of Lepr antagonists that cannot cross blood brain barrier^52^ for enhancing mobilization would be highly promising.

## Methods

### Mouse models

All animal studies were approved by the Institutional Animal Care and Use Committee (IACUC) at North Dakota State University. All experiments were carried out in accordance with guidelines and regulations approved by IACUC. Male C57Bl/6 NHsd (wild type) (Harlan Laboratories), and Lepr knockout mice (heterozygous (Lepr^+/−^) or lean-control, and homozygous (Lepr^−/−^) or obese-diabetic (db/db)) (Jackson Labs, Bar Harbor, ME, USA) were used in this study. All mice were maintained on a 12-hour light-dark cycle with food and water *ad libitum*.

Type 1 diabetes was induced by streptozotocin (STZ) in male C57Bl/6 NHsd mice^53^. Mice were treated with STZ intraperitoneally at a dose of 40 mg/kg body weight for five consecutive days. Mice with blood glucose of 250 mg/dL or higher were considered diabetic. Blood glucose levels were determined by glucose test strips (Clarity Advanced). Mice were used at a shorter (10–12 weeks) or longer (≥20 weeks) duration of diabetes. STZ and db/db mice showed blood glucose levels of 371 ± 16 mg/dL and 450 ± 17 mg/dL, respectively.

### Experimental protocols

Mobilization of bone marrow cells was induced by G-CSF (Peprotech, or Neupogen, Amgen) at a dose of 125 μg/kg, s.c., twice a day for 4 days^54^ or a single dose of AMD3100 (Tocris) (5mg/kg, s.c.)^34^. A single dose of AMD3100 induced mobilization in an hour following the treatment.

PEGylated super leptin antagonist, PESLAN-1 (Protein Laboratories Rehovot Ltd.) was administered at a dose of 10 mg/kg, s.c., every alternate day for 13 or 17 days, as needed^24^,^55^. The control group received the vehicle, double distilled water of pH 8–9. To evaluate the effect of PESLAN-1 on mobilization, G-CSF treatment was started on day-14 of PESLAN-1 treatment. G-CSF mobilization was evaluated 12 hours after the last dose of G-CSF, on day-18, following the administration of last dose of PESLAN-1 on day-17. In case of AMD3100, treatment was given on day-14, following the last dose of PESLAN-1 on day-13, and mobilization was evaluated one hour following AMD3100 administration.

Recombinant leptin (Sigma-Aldrich) was administered intraperitoneally at a dose of 600 μg/kg^56^ and the control group received normal saline, once a day, for five days. To evaluate the effect of leptin on mobilization, G-CSF treatment was started on day-2 of leptin treatment, and mobilization was evaluated 12 hours after the last dose of G-CSF on day-6. Mobilization by AMD3100 was evaluated on day-6, as described above.

### Preparation of cells and flow cytometric analysis

Peripheral blood was collected from mice under light isoflurane (Isoflurane, Terrell, USA) anesthesia in EDTA-coated microcentrifuge tubes. Red blood cells were lysed by using 0.8% ammonium chloride containing 2 mM EDTA, incubated in ice-cold conditions for 10 minutes. Then, the cell suspension washed with 1× Phosphate Buffered Saline (PBS) (Corning Cellgro) and centrifugation at 180 g once, followed by at 120 g twice. Cell pellet was re-suspended, and total WBCs were counted by using Neubauer Chamber.

**Figure 8:**
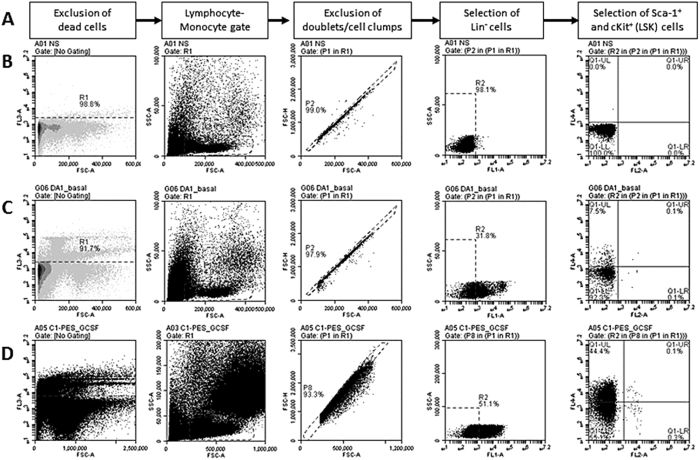
Flow cytometric enumeration of LSK cells in mouse peripheral blood. **(A)** Gating strategy for the selection of LSK cells. (**B–D)** Representative dot plots of flow cytometric analysis: (**B**) a sample treated with isotype controls, or (**C**) with fluorescent-conjugated antibodies, and (**D**) a sample with G-CSF-mobilized cells. Sequential gating involves exclusion of dead cells, stained by 7-AAD, selection of cells in lymophocyte/monocytes, exclusion of doublets or cell clumps, selection of Lineage negative (Lin^−^) cells (exclusion of differentiated cells) and then selection of c-Kit and Sca-1 dual-positive cells (LSK).

Flow cytometric enumeration of LSK cells was carried out as described before^57^. Briefly bone marrow cells that were obtained as described above were resuspended in cell staining buffer (Biolegend) and treated with 0.5 μL of antimouse CD16/32 (TruStain fcX, BioLegend), followed by the fluorescent-conjugated antibodies, Lineage cocktail-FITC, Sca-1-APC, and cKit-PE, as required. Samples were incubated for 45 minutes at 4 °C in dark. 7-AAD (BD Pharmingen) was added to detect dead cells. A non-stain sample, with isotype controls was always included in the protocol. Flow cytometry was carried out by using Accuri C6 flow cytometer and the data was analyzed off-line. Schematic of gating strategy with representative dot plots for flow cytometric enumeration of LSK cells is shown in Fig. 8.

### Colony Forming Unit (CFU) assay

Progenitor cells were enumerated by CFU assay. This was carried out by using Methocult assay kit (GF M3434, StemCell Technologies) that induces formation of all lineages of blood cells, according to the manufacturer’s instructions. Colonies were enumerated by using a counting grid (StemCell Technologies).

### Isolation of bone marrow Lin^−^cKit^+^ (LK) cells

**Figure 9:**
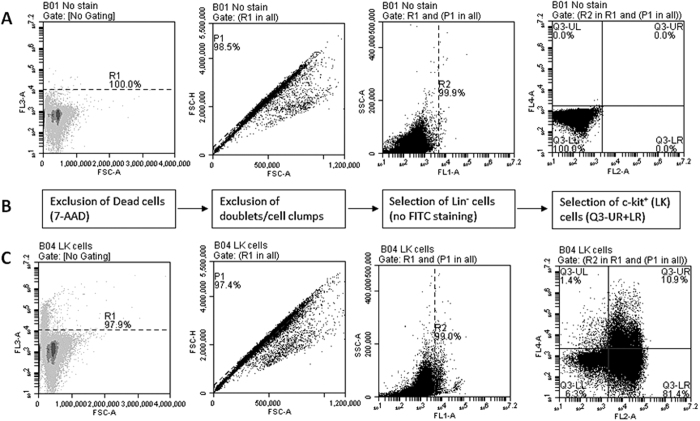
Flow cytometric evaluation of the purity of enriched LK cells. Representative dot plots of flow cytometric analysis of LK cells that were enriched by immunomagnetic selection as described in the text: (**A**) Sample treated with isotype controls, and (**C**) sample treated with fluorescent-conjugated antibodies. (**C**) Gating strategy for the selection of LK cells. Sequential gating involves exclusion of dead cells, stained by 7-AAD, exclusion of doublets or cell clumps, selection of Lineage negative (Lin^−^) cells (exclusion of differentiated cells) and then selection of c-Kit^+^ cells (LK). Note the presence a small population of LK cells that are positive Sca-1, and Lin^−^ cells that are not expressing Sca-1 or c-Kit.

Bone marrow was flushed from femur and tibia, and suspended in phosphate buffered saline. RBCs were removed by ammonium chloride-lysis solution. Lineage-negative (Lin^−^) population of cells was isolated by negative selection by using immunomagnetic enrichment kit (StemCell Technologies) as per the manufacturer’s instructions. Briefly, the mononuclear cells were suspended in the recommended medium and incubated with specific antibodies for labeling unwanted nonhematopoietic stem/progenitor cells which include CD5, CD11b, CD19, CD45R, 7–4, Ly-6G/C (Gr-1), and TER119-expressing cells. Antibody-treated cells were then labeled by Tetrameric Antibody Complexes that recognize biotin and dextran-coated magnetic particles. Unwanted labeled cells were then separated by using an EasySep™ magnet. Thus the desired Lin^−^ cells were obtained by negative selection. These cells were then enriched for cKit^+^ cells by using positive immunomagnetic selection kit (StemCell Technologies). LK cells were plated in RPMI1640 (GE Healthcare) in U-bottom, 96-well plate at a low density of 2 × 10^4^ cells/150 μL per well, until they are used for proliferation or migration assay in less than 24 hours following isolation. Representative flow cytometric dot plots evaluating the purity of LK cells enriched by this method is shown in Fig. 9.

### Proliferation assay

Proliferation of LK cells was evaluated as described earlier^57^ by determining BrdU incorporation using a kit (Cell Proliferation ELISA, Roche Bioscience) as per the manufacturer’s instructions. The assay was performed by using 10,000 cells per condition, and each sample was tested in duplicate. Cells were plated in RPMI1640 with or without drug treatments, and the proliferation was evaluated after 48 hours. Absorbance was quantified by using Spectramax plate reader. Proliferation was expressed as fold increase relative to the effect of mitomycin (1μM), which inhibits proliferation.

### Migration assay

Migration of LK cells was evaluated as described before^39^, by using QCM™ Chemotaxis cell migration assay kit (EMD Millipore) as per the manufacturer’s instructions. Assay was carried out by using 20,000 cells per treatment in a basal medium, HBSS (Mediatech, Inc.) and each sample was tested in duplicate. Cells were allowed to migrate in response to the treatments for five hours, and the response was recorded as Arbitrary Fluorescence Units (AFUs) and presented as percent increase over untreated control of the same group.

### Hind-limb ischemia (HLI) and blood flow imaging

Surgery was carried out in mice under isoflurane anesthesia. A 1–1.5 cm incision was made in the skin in the left inguinal area. Femoral artery was exposed and ligated with 7-0 silk suture as described by Niiyama *et al.*^58^, and the incision was closed with 7-0 vicryl suture. Hind-limb blood-flow was determined by imaging the flux (blood × area^−1 ^× time^−1^) of blood by using Laser Doppler imaging system (Moor Instruments Inc.) under isoflurane anesthesia. Blood flow recovery in the ischemic limb is expressed relative to the mean blood flux in the contralateral non-ischemic limb. Mobilization of LSK cells was determined before and at different time points following HLI by flow cytometry as described above.

Biochemical analysis of selected factors, SDF, VEGF, IGF1 and IGFBP-3, in plasma was carried out by Luminex multiplex protein array (Assaygate, Inc., Ijamsville, MD).

### Data analysis

Results are expressed as Mean ± S.E.M. Number of experiments ‘n’ indicates the number of mice used in the experimental group. Experimental groups or treatments were compared for significant difference by either Student’s ‘t’-test or ANOVA with Bonferroni’s post-test, as applicable, by using GraphPad Prism software (GraphPad Software, Inc.). Treatment groups were considered significantly different if P < 0.05.

## Additional Information

**How to cite this article**: Vasam, G. *et al.* Impaired Mobilization of Vascular Reparative Bone Marrow Cells in Streptozotocin-Induced Diabetes but not in Leptin Receptor-Deficient db/db Mice. *Sci. Rep.* **6**, 26131; doi: 10.1038/srep26131 (2016).

## Figures and Tables

**Table 1:** Plasma levels of endogenous factors that are known to sensitize bone marrow cells for mobilization.

	C57Bl/6 NHsd	STZ-Diabetes	Lean-Control	db/db
N	8	7	6	5
SDF (pg/ml)	830 ± 106	885 ± 113	621 ± 61	608 ± 92
VEGF (pg/ml)	54 ± 8	65 ± 12	17 ± 5	16 ± 3
IGF-1 (ng/ml)	45 ± 3	41 ± 2	49 ± 9	81 ± 5*
IGFBP-3 (ng/ml)	208 ± 13	175 ± 16	182 ± 11	225 ± 19

*P < 0.001 compared to other three groups of mice (One-way ANOVA, Bonferroni post-test).
